# Accrediting Neurology Fellowships Accelerates Subspecialization

**DOI:** 10.3389/fneur.2013.00094

**Published:** 2013-07-23

**Authors:** Trent S. Hodgson, James R. Brorson, Agnieszka A. Ardelt, Rimas V. Lukas

**Affiliations:** ^1^Pritzker School of Medicine, University of Chicago, Chicago, IL, USA; ^2^Department of Neurology, University of Chicago, Chicago, IL, USA; ^3^Section of Neurosurgery, Department of Surgery, University of Chicago, Chicago, IL, USA

**Keywords:** education, neurology, fellowships, certification, accreditation, neurocritical care, vascular neurology, subspecialization

## Introduction

In the 2011 American Academy of Neurology Residents Survey 86% of American neurology residents planned to complete a fellowship after their residency, up from estimates of 78% in 2008 and 74% in 1996 (1–3). There has been an increase in the types of subspecialty fellowship programs and, importantly, the available certifications. To understand the impact of this increasing accreditation one can compare two fields that overlap in the care of patients with acute ischemic or hemorrhagic stroke: Vascular Neurology, which is certified by the American Board of Psychiatry and Neurology (ABPN), and Neurocritical Care, which is certified by the United Council for Neurologic Subspecialties (UCNS).

Within the United States and Canada, the ABPN and UCNS are the primary accrediting bodies of neurology fellowships. These organizations accredit fellowship programs that have applied and met predetermined standards. They also create their own exams and administer certification to qualified physicians who pass. The ABPN was founded in 1934 and the UCNS was founded in 2003 (Table A1 in Appendix). The organizations have similarities, but one difference is the UCNS's interest in serving small subspecialties (4). This is significant for emerging subspecialties within neurology as the importance assigned to physician certifications by patients, employers, and other physicians will likely continue to grow.

Changes in education and accreditation often occur after changes in practice have already occurred. Neurocritical Care has emerged as a new subspecialty over the past 30 years based on perceptions and evidence that neurologists with expertise in this area improved patient care (5). Fellowships in Neurocritical Care have existed for a number of years, but it is unclear how the creation of a UCNS Neurocritical Care certification in 2007 affected the neurology community's perception of Neurocritical Care as a subspecialty distinct from Vascular Neurology and General Neurology. Here we present data that the accreditation of Vascular Neurology and Neurocritical Care fellowships appears to have accelerated the divergence of these subspecialties.

## Abpn and UCNS Diplomates

Analysis of publicly available lists of Vascular Neurology diplomates from the ABPN and Neurocritical Care diplomates from the UCNS can provide a clearer understanding of subspecialization trends in Neurology. Both lists include the full legal name, degrees held, and city of residence at the time of certification. This allows for determination of which diplomates received both certifications. Of note, these lists include only those who have passed the exam and received certification, not those who sat for the exam but did not pass. The UCNS does not publish passing rates, but the ABPN reports between 92.3 and 94.7% passing from 2008 to 2011.

## Vascular Neurology and Neurocritical Care

**Figure 1:**
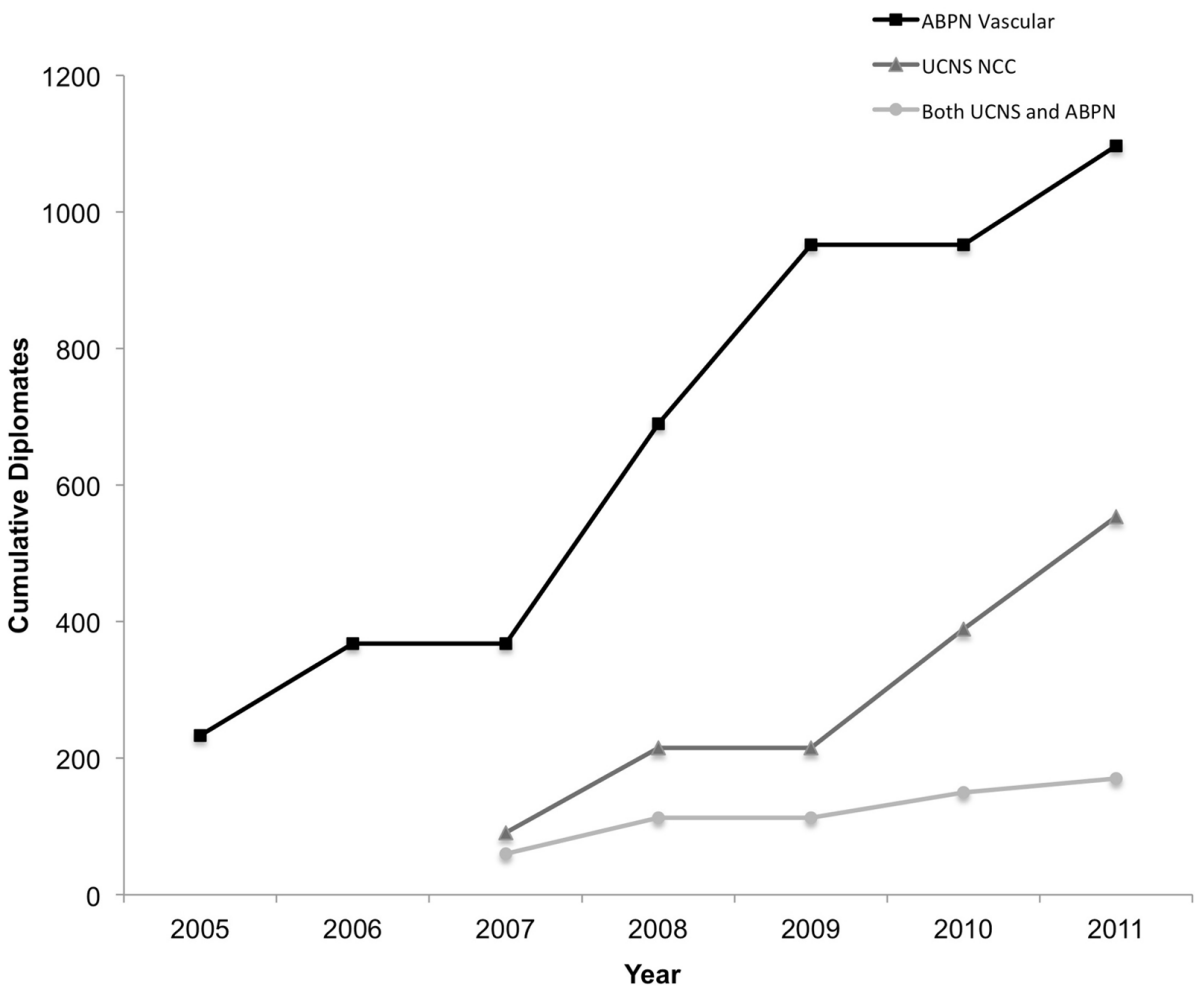
**Cumulative number of diplomates by year and certification(s) held**. NCC, Neurocritical Care.

The ABPN first issued certifications in Vascular Neurology in 2005 and the UCNS began issuing Neurocritical Care certifications in 2007 (Figure 1). In their inaugural years, there were 234 Vascular Neurology diplomates compared to 91 Neurocritical Care diplomates. Through 2011, 1098 ABPN Vascular Neurology certifications have been issued and publically listed, almost twice as many as the 554 UCNS Neurocritical Care certifications. The flat sections in the cumulative totals demonstrate that both exams are offered only 2 out of every 3 years.

**Figure A1:**
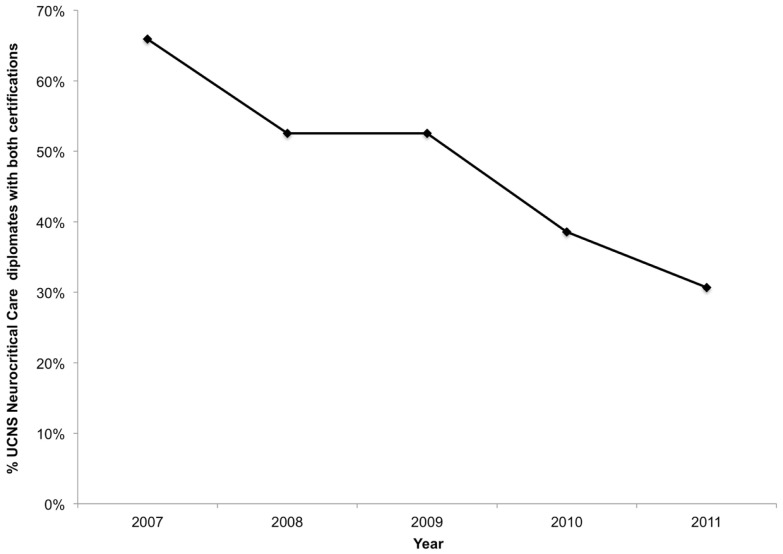
**Cumulative percentage of UCNS diplomates who also hold ABPN Vascular Neurology certification by year of UCNS certification**.

The percentage of individuals who hold both certifications was initially high but has since declined (Figure A1 in Appendix). Of the 91 physicians who received their UCNS Neurocritical Care certification in 2007, 60 (65.9%) have also received ABPN certification in Vascular Neurology. This is significantly more than the 20 out of 165 UCNS Neurocritical Care diplomates (12.1%) in 2011 that also hold ABPN Vascular Neurology certification (*p* < 0.001).

**Figure A2:**
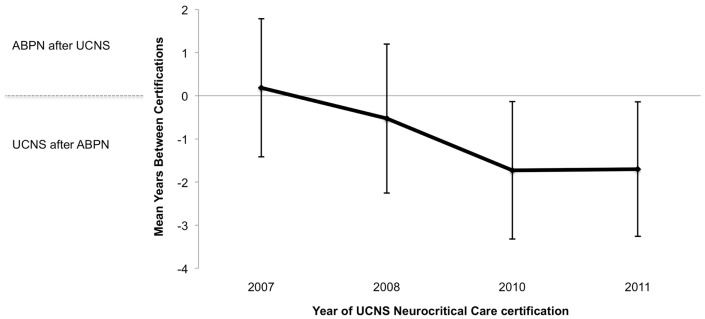
**Average number of years between receiving each certification in individuals with both certifications listed by year of UCNS certification**. For each individual, the difference is calculated as year of ABPN certification minus year of UCNS certification. Error bars are ± 1 SD.

In addition, fewer physicians are going on to obtain their ABPN Vascular Neurology certification after they have received UCNS Neurocritical Care certification (Figure A2 in Appendix). Of those with both certifications, 61.7% of 2007 UCNS Neurocritical Care diplomates went on to obtain their ABPN Vascular Neurology certification in the same calendar year or later than their UCNS Neurocritical Care certification. This decreased to 35.0% of 2011 UCNS diplomates (2007 vs. 2011, *p* = 0.01).

## Discussion

The practice of Neurocritical Care has defined itself over the past several decades. This differentiation is a gradual, ongoing process led by changes in clinical realities, technical advances, and a dedicated journal (6, 7). In addition to these primary influences, we present evidence that UCNS certification of preexisting Neurocritical Care fellowships may have accelerated the subspecialization of Neurocritical Care.

Multiple factors contribute to nearly twice as many ABPN Vascular Neurology certifications as UCNS Neurocritical Care certifications being issued to date. The practice of Vascular Neurology is older, taught in every residency, and more exclusively the realm of neurologists. Of the 7188 practicing US neurologists who responded to the 2008 American Academy of Neurology census, 37.0% selected cerebrovascular disease as an area of practice focus compared to 9.2% who listed critical care. Thus, it would be expected that there is a higher absolute number of individuals practicing Vascular Neurology who would desire certification. Additionally, a significant portion of neurology residents do not have required exposure to Neurocritical Care during their training and in turn may not seek additional training in this subspecialty.

Whatever the field's initial interest, there was a substantial degree of overlap in individuals receiving both certifications (65.9% of 2007 UCNS Neurocritical Care diplomates). However, since 2007, the percentage of individuals holding both certifications decreased significantly as did the percentage of individuals who went on to obtain their ABPN Vascular Neurology certificate the same year or later than their UCNS Neurocritical Care certification. This likely reflects the continued emergence of Neurocritical Care as a distinct subspecialty and may reflect the increased stature of the UCNS and its certifications.

The end of grandfathering periods, where qualified physicians who trained before accreditation was available are allowed to take the certification exam, will further impact the divergence of these subspecialties. The ABPN Vascular Neurology grandfathering period was from 2005 to 2009 and the UCNS certification in Neurocritical Care grandfathering period is from 2007 to 2013. It would be expected that the proportion of diplomates with both certifications would decrease with the end of the ABPN Vascular Neurology grandfathering period in 2009. However, the rate of decline of individuals with both certifications as a proportion of all Neurocritical Care diplomates before and after 2009 is similar suggesting that this alone cannot explain our observations.

## Future of Subspecialization

There are pros and cons to increasing subspecialization within neurology (8–11). Increased subspecialization can facilitate clinical and translational research as well as improved patient care. However it may artificially restrict the scope of practice of neurologists who already completed their training, trained in another subspecialty, or completed an unaccredited fellowship. The impact will likely vary between different practice types, especially academic and non-academic settings. It will also depend on employers’ perception of the value of certification as well how certification, or the lack thereof, affects individual physicians’ comfort level practicing in the ICU.

It will be important to monitor both economic and geographic growth trends to better understand the impact of increasing accreditation of neurology fellowships. This data should then be used to prevent unintended consequences such as fragmentation of care, over-referral, and a geographic maldistribution of subspecialists. In this way, the decision to pursue fellowship training can be based on the desire to gain expertise that improves patient care more than any secondary gains.

## Conclusion

Accreditation of subspecialty training programs and certification of their graduates has served as a catalyst accelerating the ongoing subspecialization of Neurology. Our discussion of the progressive differentiation of the related subspecialties of Vascular Neurology and Neurocritical Care supports this conclusion. While our discussion focused on fellowship training in the U.S. and Canada these trends are likely to influence neurology fellowship training throughout the world. Going forward, it is important to understand the impact of accreditation on patient care, reimbursements, and the neurology workforce and to use this knowledge to guide future decisions on accreditation. As any decision will carry benefits and drawbacks, accrediting bodies including the ABPN and UCNS should maintain a transparent process whose primary goal is to improve the treatment of patients with neurologic disease.

## Appendix

**Table A1:** **ABPN and UCNS specialties and subspecialties**. The year each was initially accredited is listed in parentheses.

	American Board of Psychiatry and Neurology	United Council for Neurologic Subspecialties
Founded	1934	2003
Primary certifications	Psychiatry, Neurology, Child Neurology	n/a
Accredited subspecialties*	Clinical Neurophysiology (1989)	Behavioral Neurology and Neuropsychiatry (2004)
	Pain Medicine (1998)	Clinical Neuromuscular Pathology (2005)
	Neurodevelopmental Disabilities (1999)	Headache Medicine (2005)
	**Vascular Neurology (2003)**	Neuro-Oncology (2005)
	Neuromuscular Medicine (2005)	Neuroimaging (2005)
	Sleep Medicine (2005)	**Neurocritical Care (2005)**
	Hospice and Palliative Care (2006)	Autonomic Disorders (2007)
	Epilepsy (2011)	Geriatric Neurology (2007)
	Brain Injury Medicine (2011)	Neural Repair and Rehabilitation (2010)

**Listed by year of approval, which is typically 2 years before the first exam is offered. Five ABPN Psychiatry-only subspecialties are not listed*.
